# Supplementary material for: Mandibular morphology clarifies phylogenetic relationships near the origin of crown birds
Source: BMC Ecol Evol. 2025 Dec 25;26:11. doi: 10.1186/s12862-025-02487-4 (PMC12849110; doi:10.1186/s12862-025-02487-4)
Supplement: Supplementary file 1 — Supplementary material [file 12862_2025_2487_MOESM1_ESM.docx]

Supplementary Materials

| **Taxon** | **Specimen Number** |
| --- | --- |
| **Palaeognathae** | |
| *Struthio camelus* (subadult) | UMZC uncatalogued |
| *Dromaius novaehollandiae* (immature) | UMZC uncatalogued |
| *Dromaius novaehollandiae* (adult) | UMZC 362 |
| *Casuarius casuarius* (adult) | UMZC 372.c |
| *Rhea americana* (adult) | FMNH B 339616 |
| *Tinamus solitarius* (immature) | UMZC uncatalogued |
| *Eudromia elegans* (adult) | UMZC 404.E |
| *Crypturellus tataupa* (adult) | UMZC uncatalogued |
| **Anseriformes** | |
| *Chauna chavaria* (immature) | UMZC 12.Anh.2.a.3 |
| *Chauna chavaria* (adult) | OUMNH 23790 |
| *Thalassornis leuconotus* (immature) | UMZC uncatalogued |
| *Thalassornis leuconotus* (adult) | UMZC 12/Ana/62/a/1 |
| *Anser fabalis* (immature) | UMZC 12/Ana/4/e/14 |
| *Anser albifrons* (adult) | UMZC 242.E |
| **Galliformes** | |
| *Megapodius pritchardii* (immature) | UMZC 14/Meg/b/g/2 |
| *Megapodius nicobariensis* (adult) | UMZC 14/Meg/f/3 |
| *Gallus gallus* (immature) | UMZC uncatalogued |
| **Neoaves** | |
| *Morus bassanus* (immature) | UMZC 10.Sul.1.a.7 |
| *Morus bassanus* (adult) | UMZC 262.C |
| *Diomedea exulans* (immature) | UMZC 9.Dio.1.h.8 |

**Supplementary Table 1.** List of neornithine specimens CT scanned and used for mandibular anatomical comparisons and figures.

**Institutional abbreviations**. **AMNH**, American Museum of Natural History, New York, USA; BHI, Black Hills Institute of Geological Research, Hill City, South Dakota, USA; **FMNH**, Field Museum of Natural History, Chicago, Illinois, USA; KUVP, Vertebrate Paleontology Division, University of Kansas Biodiversity Institute & Natural History Museum, Lawrence, Kansas, USA; **NHMM**, Natuurhistorisch Museum Maastricht, Maastricht, The Netherlands; **OUMNH**, Oxford University Museum of Natural History, Oxford, UK; **UMZC**, University of Cambridge Museum of Zoology, Cambridge, UK; **YPM**, Yale Peabody Museum, Yale University, New Haven, USA.


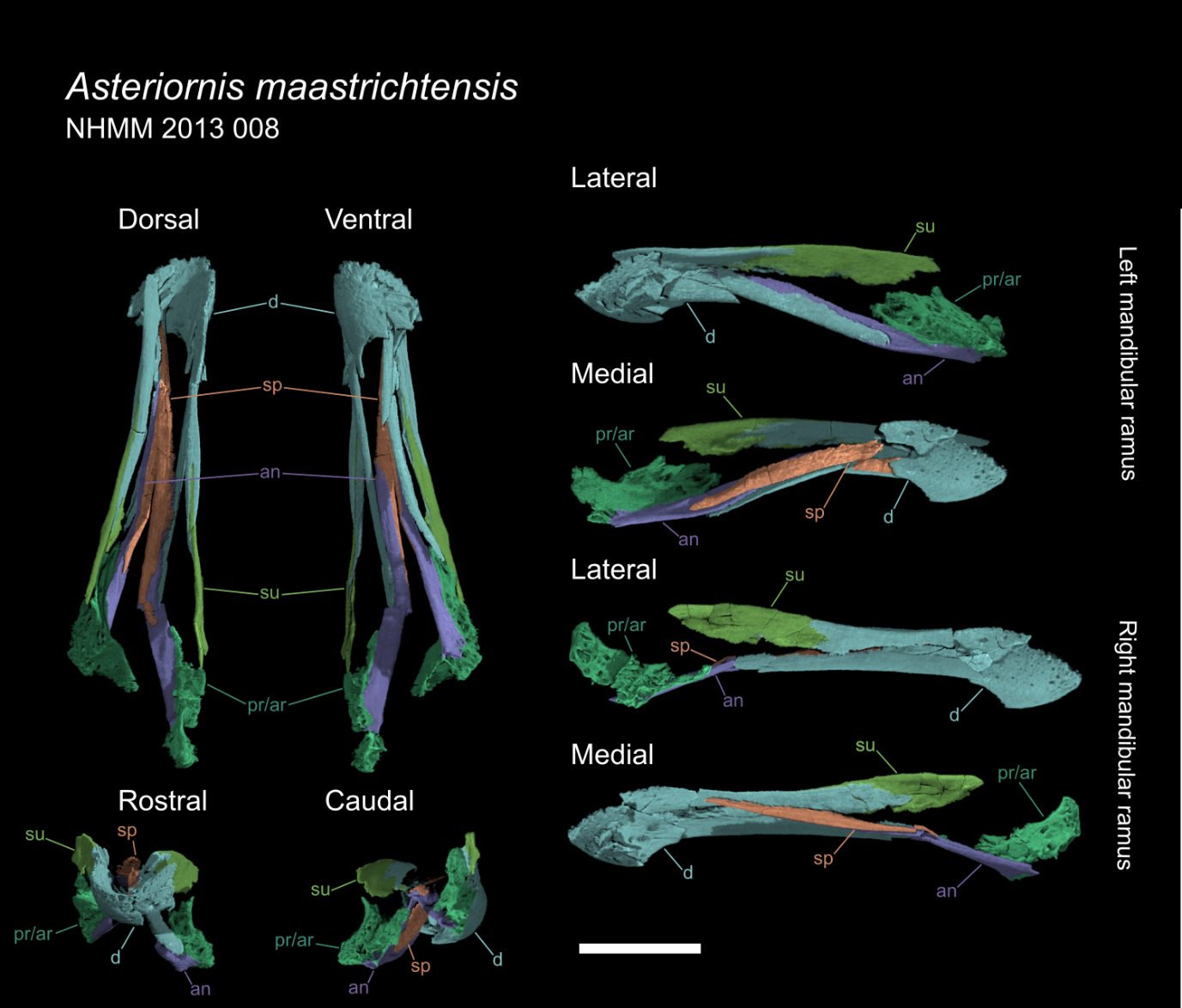


**Supplementary Figure 1.** Mandible of *Asteriornis* holotype (NHMM 2013 008), as preserved. Whole mandible shown in dorsal, ventral, rostral and caudal views. Left and right rami shown separately in medial and lateral views. Prearticular and articular could not be distinguished, displayed as prearticular/articular combined. Scale bar equals 10mm.


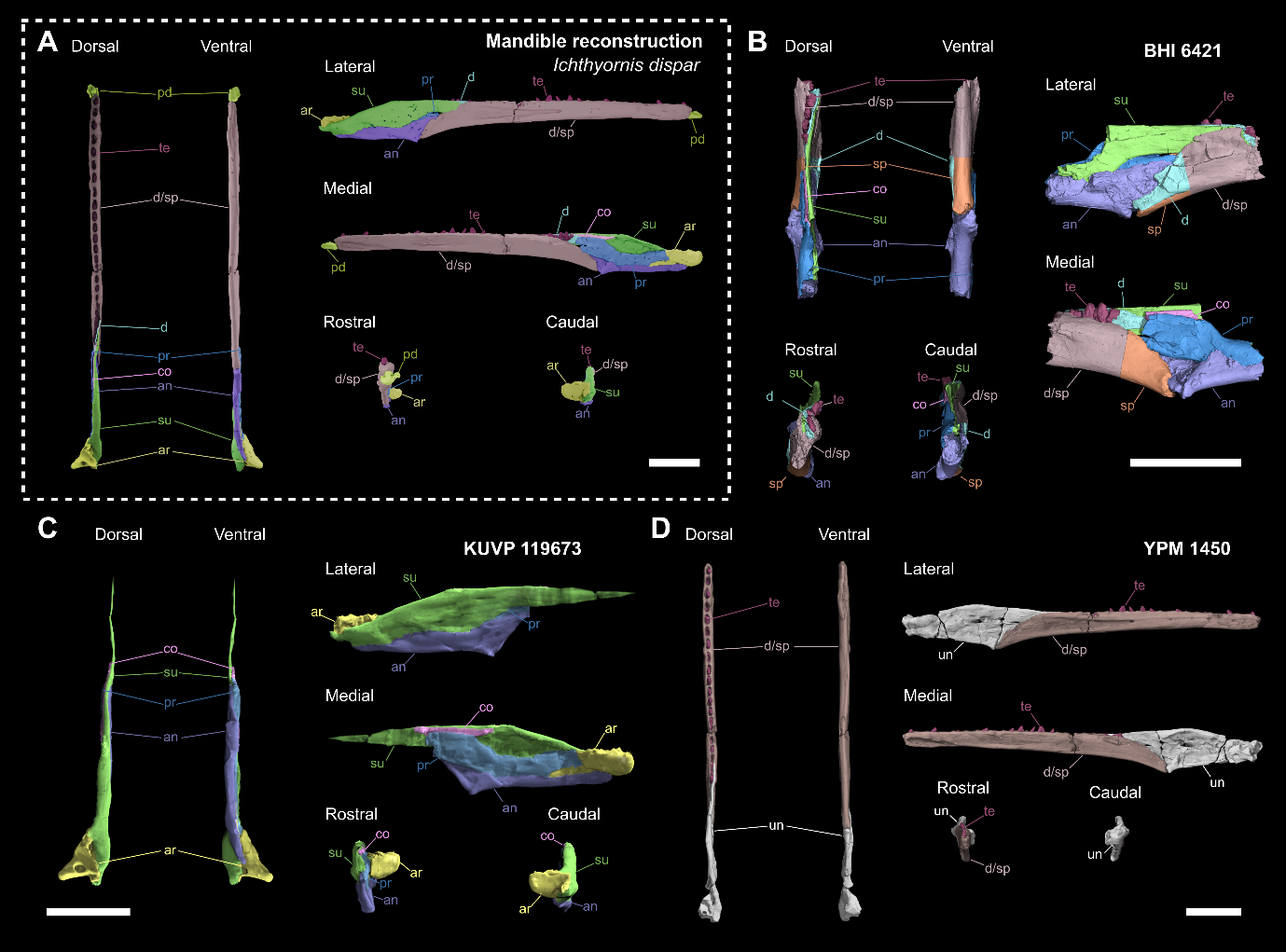


**Supplementary Figure 2.** Right mandible of *Ichthyornis dispar* shown in dorsal, ventral, rostral, caudal, lateral and medial views. A) Reconstruction of mandible composed from multiple fossil specimens (BHI 6421, KUVP 119673, YPM 1450, AMNH FARB 32773), rescaled relative to BHI 6421. B) Segmentation of mandible section from *Ichthyornis* specimen BHI 6421. C) Segmentation of mandible section from *Ichthyornis* KUVP 119673. D) Segmentation of mandible section from *Ichthyornis* YPM 1450. Scale bars equal 10mm.


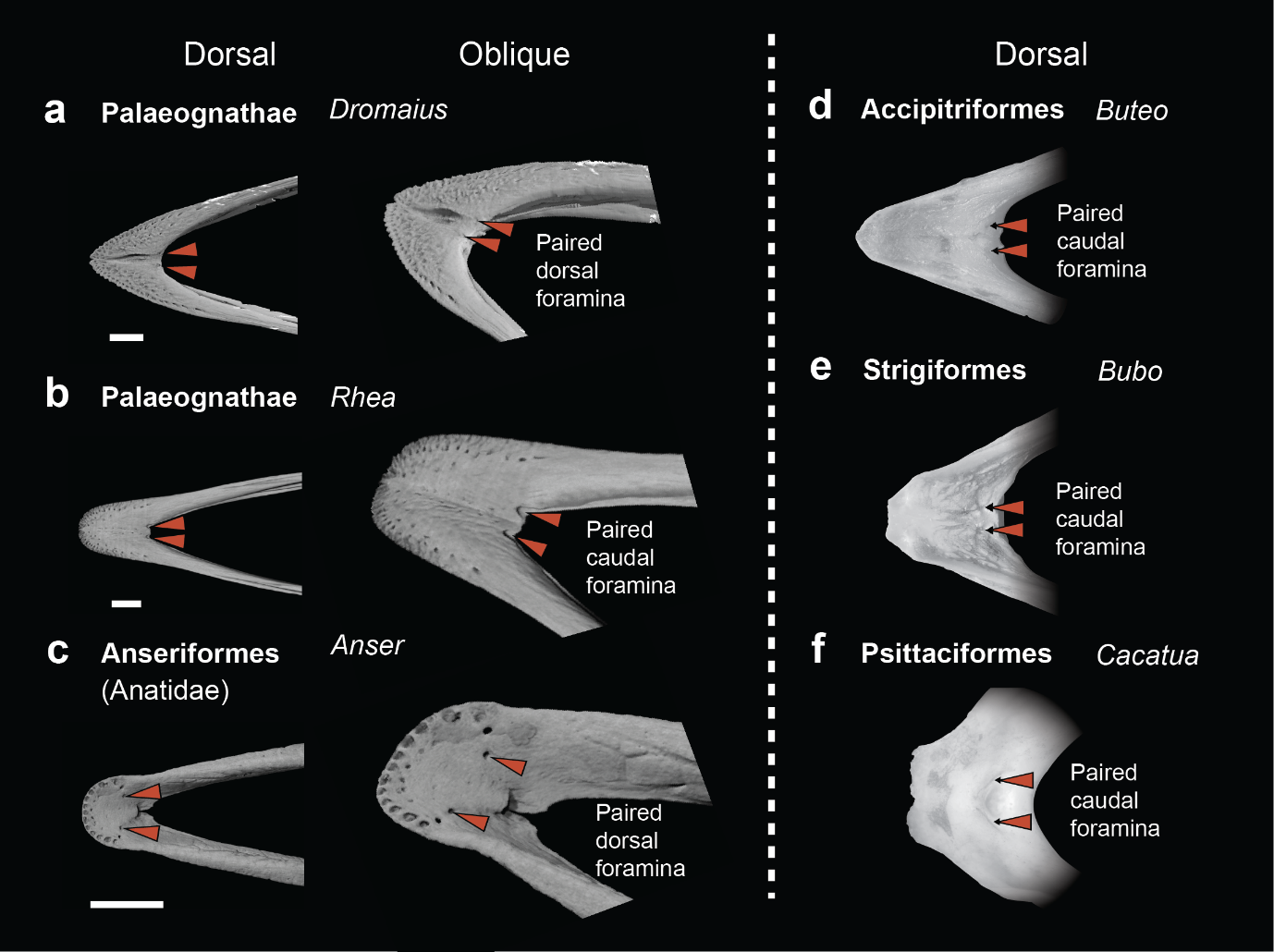


**Supplementary Figure 3.** Comparative morphology of mandibular symphyseal foramina in dorsal and (selected) dorsolateral oblique views. A) *Dromaius novaehollandiae*. B) *Rhea americana*. C) *Anser albifrons*. D) *Buteo sp*. E) *Bubo sp.* F) *Cacatua sp*.


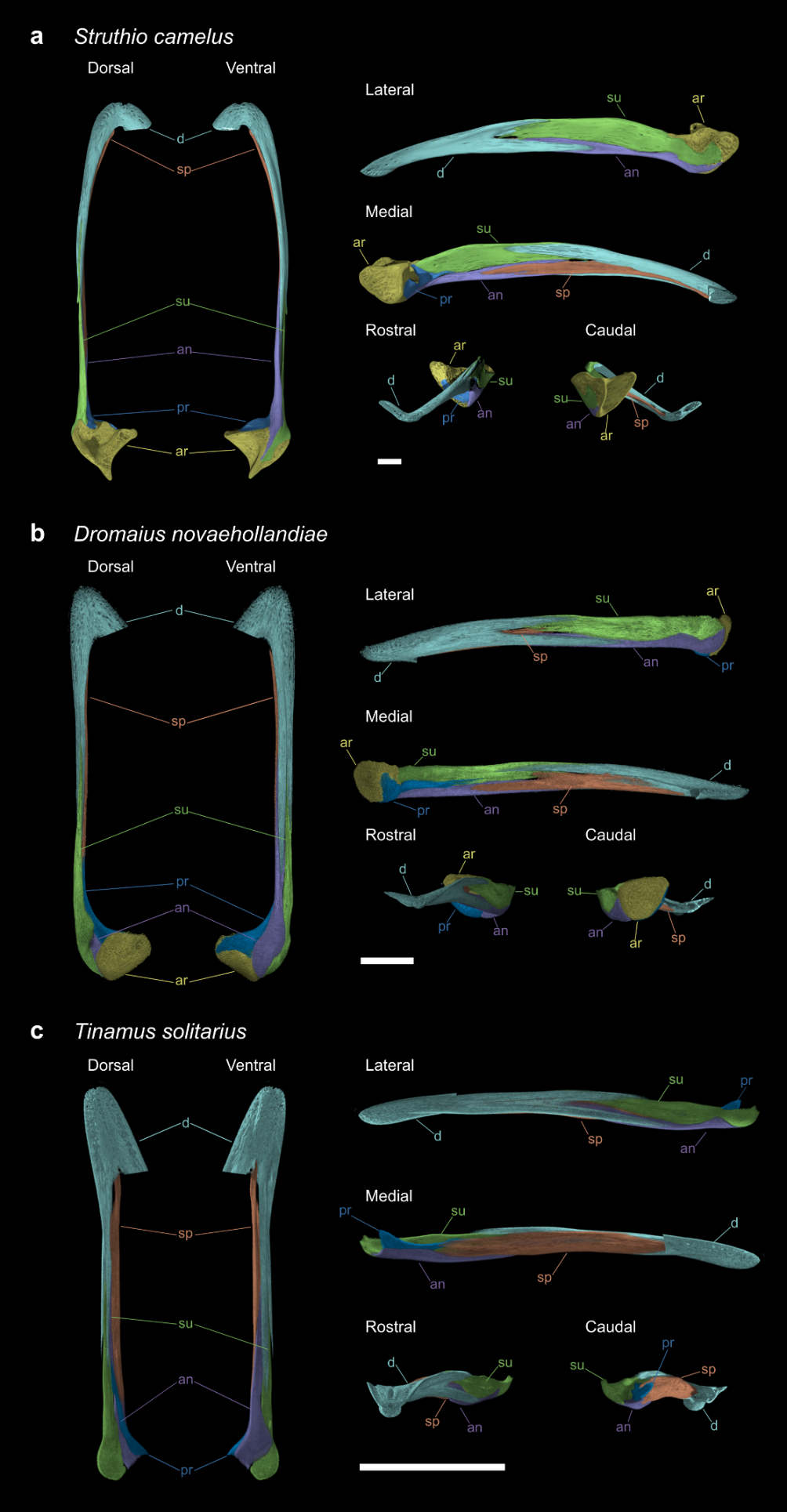
**Supplementary Figure 4.** Mandibles of juvenile palaeognaths shown in in dorsal, ventral, rostral, caudal, lateral and medial views. A) *Struthio camelus*. B) *Dromaius novaehollandiae*. C) *Tinamus solitarius-* this specimen is at an earlier ontogenetic stage and so does not exhibit an ossified articular bone. Scale bars equal 10mm.


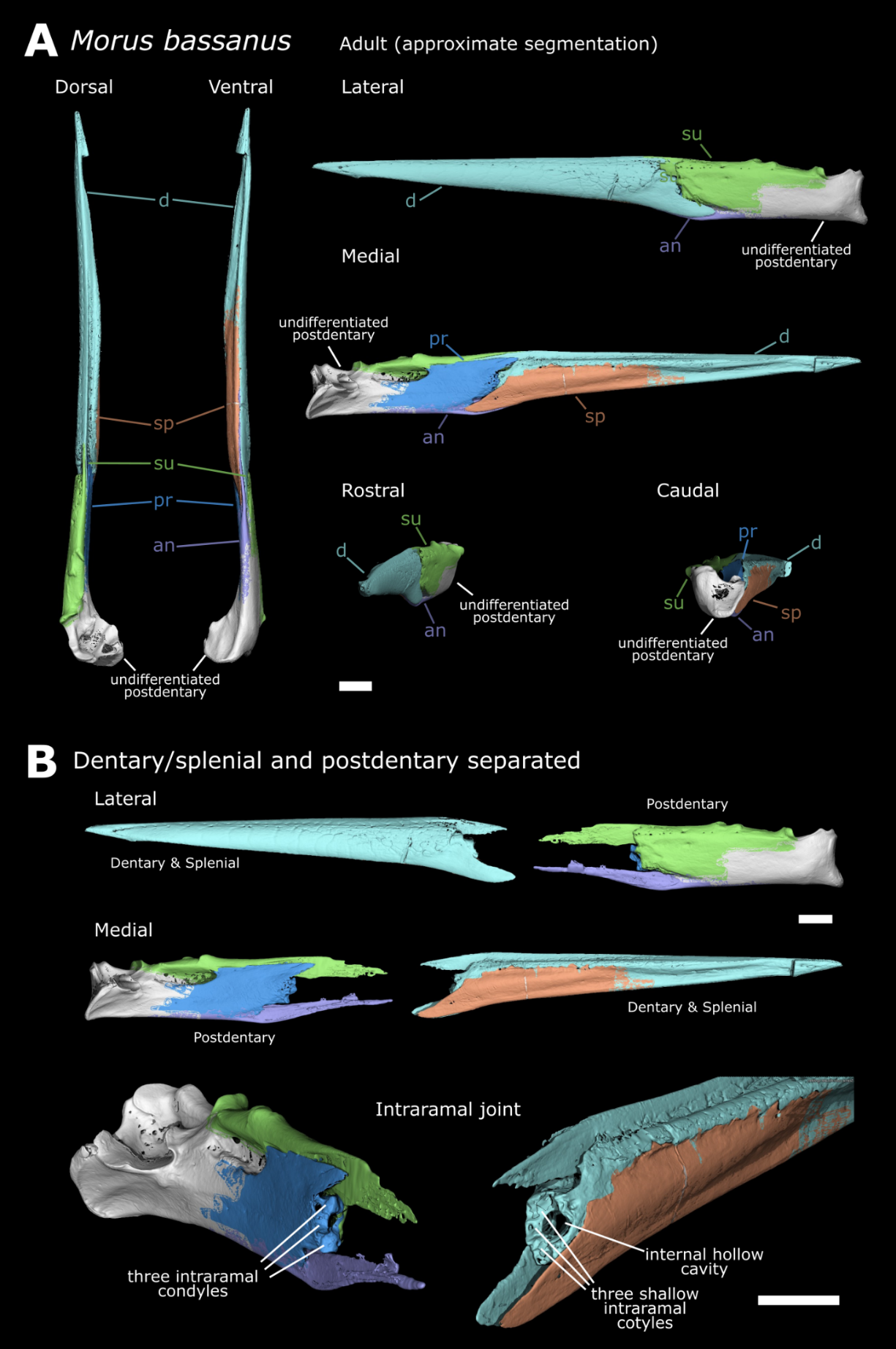
 **Supplementary Figure 5.** Mandible of adult *Morus bassanus* (Neoaves), approximately segmented. High levels of fusion in this adult specimen leads to uncertainty in parts of this segmentation; we have chosen to leave highly fused regions of the postdentary complex unsegmented. A) Whole ramus shown in dorsal, ventral, rostral, caudal, lateral and medial views. A) Mandible separated into dentary/splenial and postdentary complex, illustrating the anatomy of the intraramal joint. Ramus shown in in lateral, medial and oblique views. Scale bars equal 10mm.


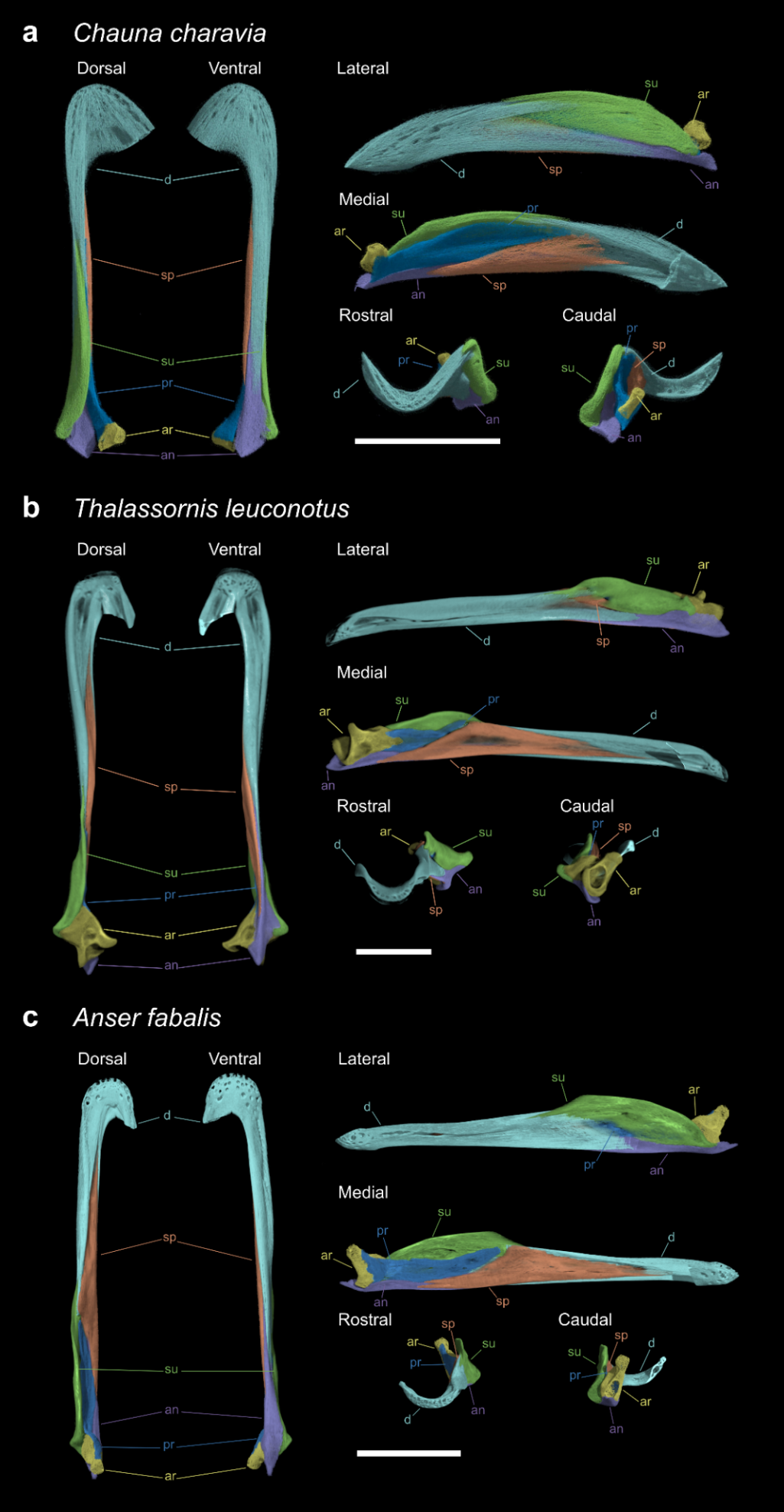
**Supplementary Figure 6.** Mandibles of juvenile anseriforms shown in dorsal, ventral, rostral, caudal, lateral and medial views. A) *Chauna charavia*. B) *Thalassornis leuconotus*. C) *Anser fabalis*. Scale bars equal 10mm.


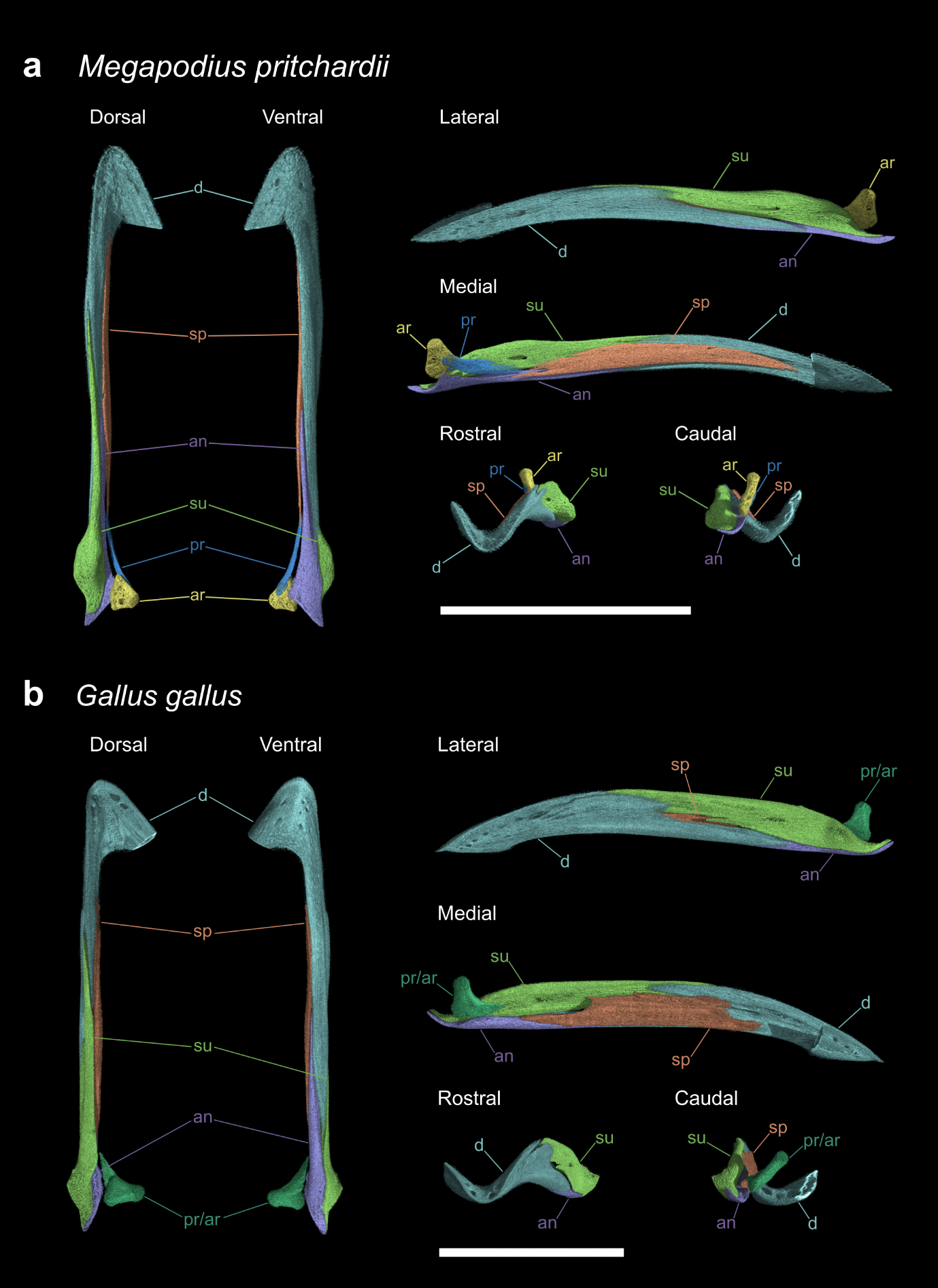


**Supplementary Figure 7.** Mandibles of juvenile galliforms shown in dorsal, ventral, rostral, caudal, lateral and medial views. A) *Megapodius pritchardii*. B) *Gallus gallus-* the prearticular and articular bones could not be distinguished in this specimen. Scale bars equal 10mm.


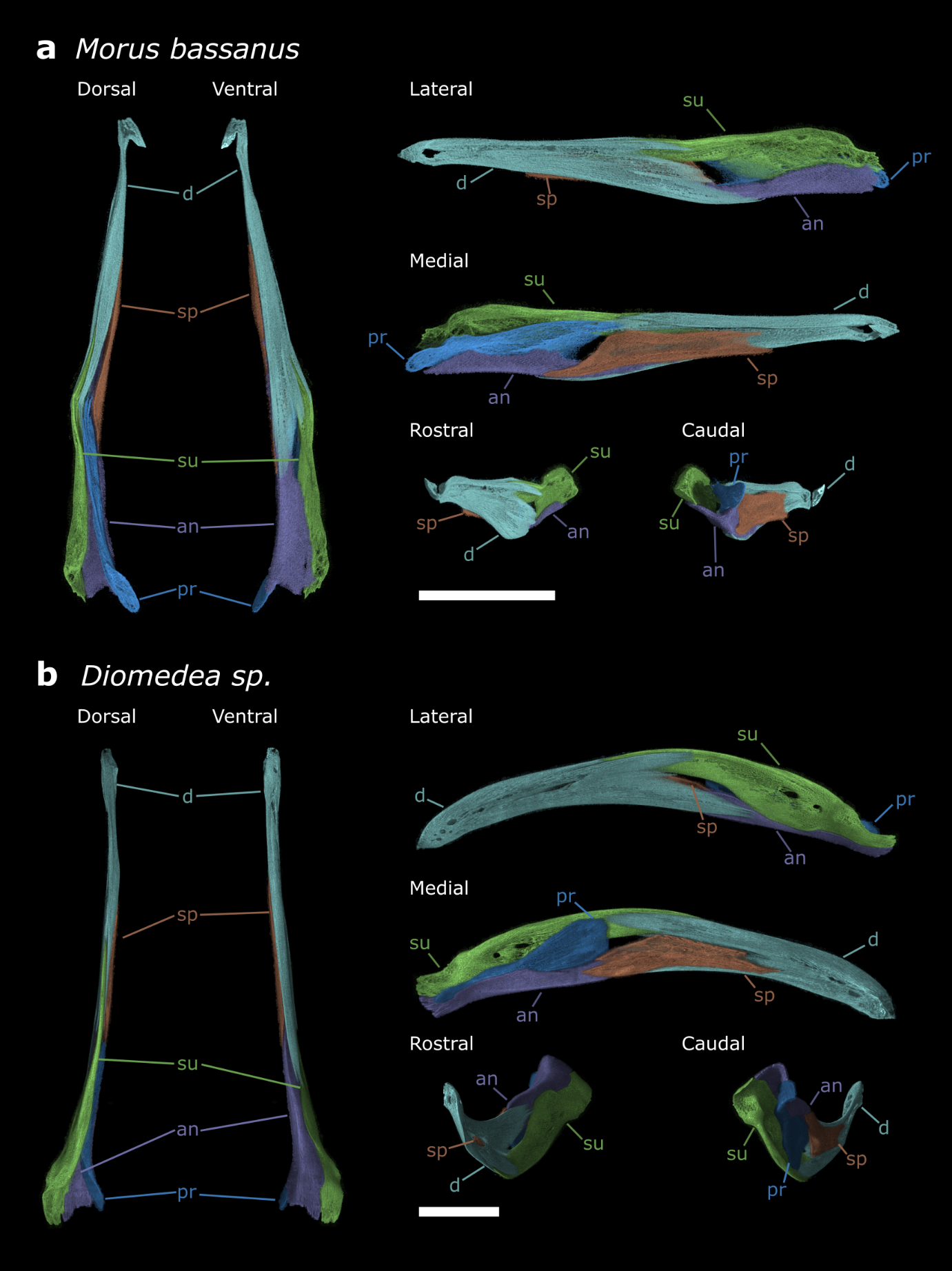


**Supplementary Figure 8.** Mandibles of juvenile neoavians with intraramal hinges shown in dorsal, ventral, rostral, caudal, lateral and medial views. A) *Morrus bassanus*. B) *Diomedea sp.* Both of these specimens are at an early ontogenetic stage and do not exhibit an ossified articular bone Scale bars equal 10mm.


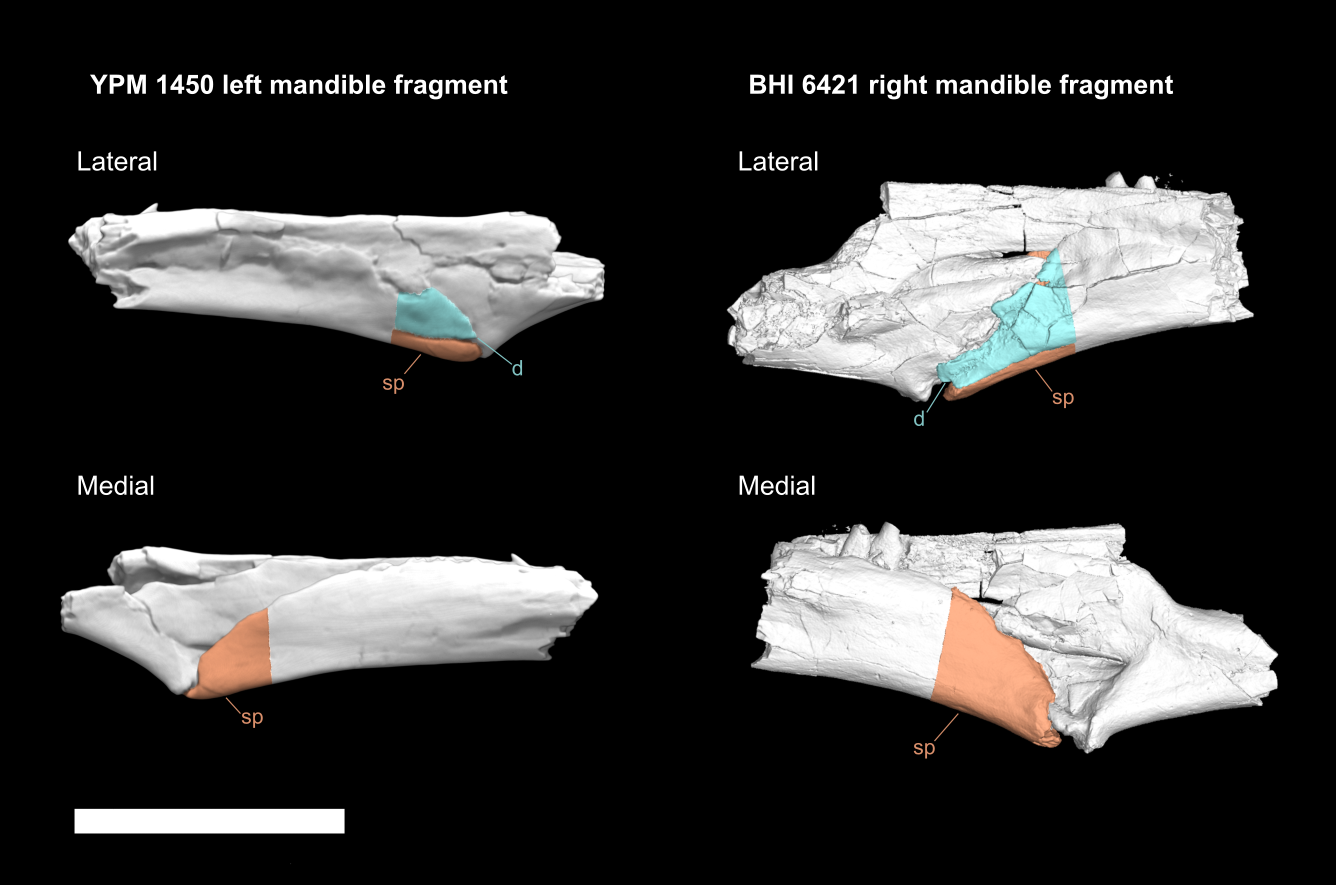


**Supplementary Figure 9.** Mandibular fragments of Ichthyornis (YPM 1450 left mandible, BHI 6421) preserving an open suture between the dentary and splenial. The open suture between the dentary and splenial at their caudal ends is described in YPM 1450 left mandible by Clarke (2005) but not figured. This same suture us observable in part of BHI 6421. Scale bar equals 10mm.


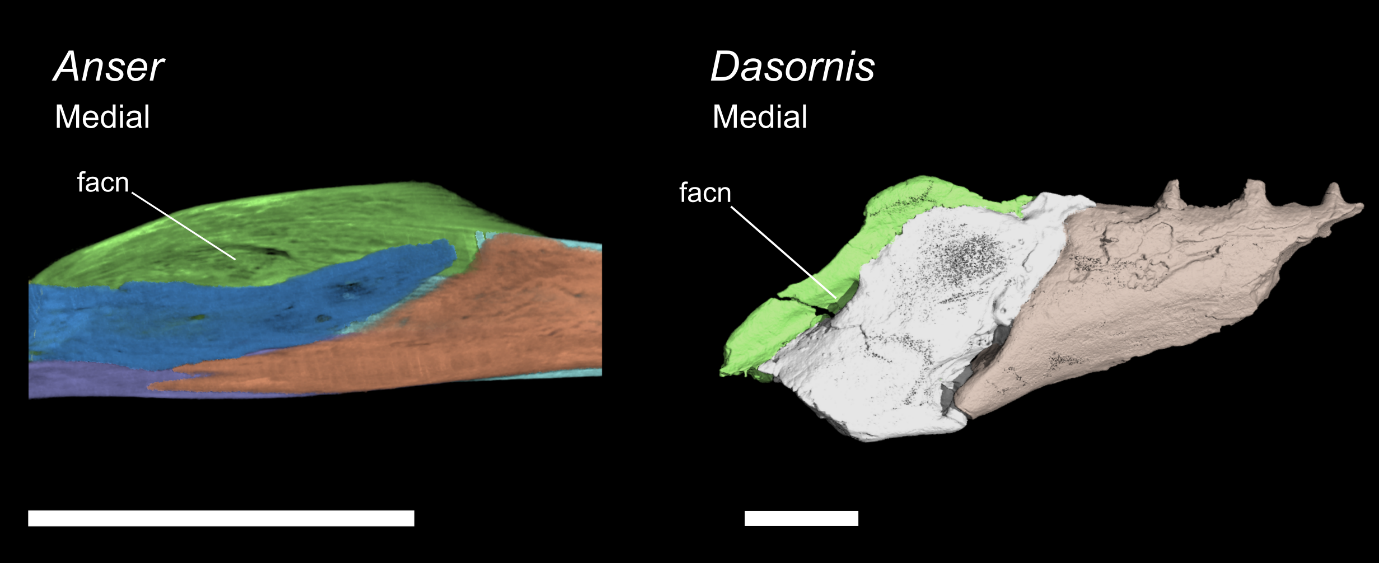


**Supplementary Figure 10.** Partial mandible of *Dasornis* and similar mandibular portion of *Anser.* Part of left ramus in medial view, illustrating the fossa adius canalis neurovascularis in these taxa. Scale bar equals 10mm.

**Phylogenetic Supplementary Material**

**File descriptions**

Phylogenetic matrix.nex: Phylogenetic matrix analyzed in the present study.

Trees 1.tre: Tree file containing results of unconstrained phylogenetic analysis (most parsimonious trees and strict consensus tree).

Trees 2.tre: Tree file containing results of phylogenetic analysis with *Asteriornis* constrained to be a total-group palaeognath (most parsimonious trees and strict consensus tree).

Analysis 1.png: Strict consensus of most parsimonious trees found by unconstrained parsimony analysis. Numbers at nodes represent bootstrap frequencies/Bremer support values.

**Additional details on phylogenetic analysis**

For our phylogenetic analyses, we modified the dataset from Torres et al. (2021) used by Benito et al. (2022). We incorporated changes to the scoring of *Vegavis* introduced by Torres et al. (2025), as well as their overall re-scoring of characters 213 and 214. Character 33 on the presence of a “well-developed tubercle on anterior surface of dorsal process” on the quadrate (identified as equivalent to the articular eminence or subcapitular tubercle by Mayr and Clarke, 2003) was re-scored from unknown to state 0 in *Lithornis* and to state 1 in *Conflicto* and *Asteriornis* following previous morphological descriptions (Nesbitt and Clarke, 2016; Tambussi et al., 2019; Field et al., 2020). Characters 137–139 were reworded to use the term “pisiform” instead of “ulnare” following studies on carpal homology by Botelho et al. (2014) and Napoli et al. (2025). Character 219 on the arrangement of tail feathers was found to have been scored erroneously for numerous taxa and re-scored based on previous morphological descriptions (Hou et al., 2004; Hu et al., 2011; O’Connor et al., 2012; O’Connor et al., 2013; Wang et al., 2014; Zhou et al., 2014; Wang et al., 2018).

Select mandibular characters were re-scored for the following taxa based on observations made during the present study:

*Vegavis*

- Character 41: 1 🡪 0

*Asteriornis*

- Character 42: 1 🡪 0
- Character 44: ? 🡪 0
- Character 48: ? 🡪 0
- Character 49: ? 🡪 0

*Gallus*

- Character 41: 1 🡪 0


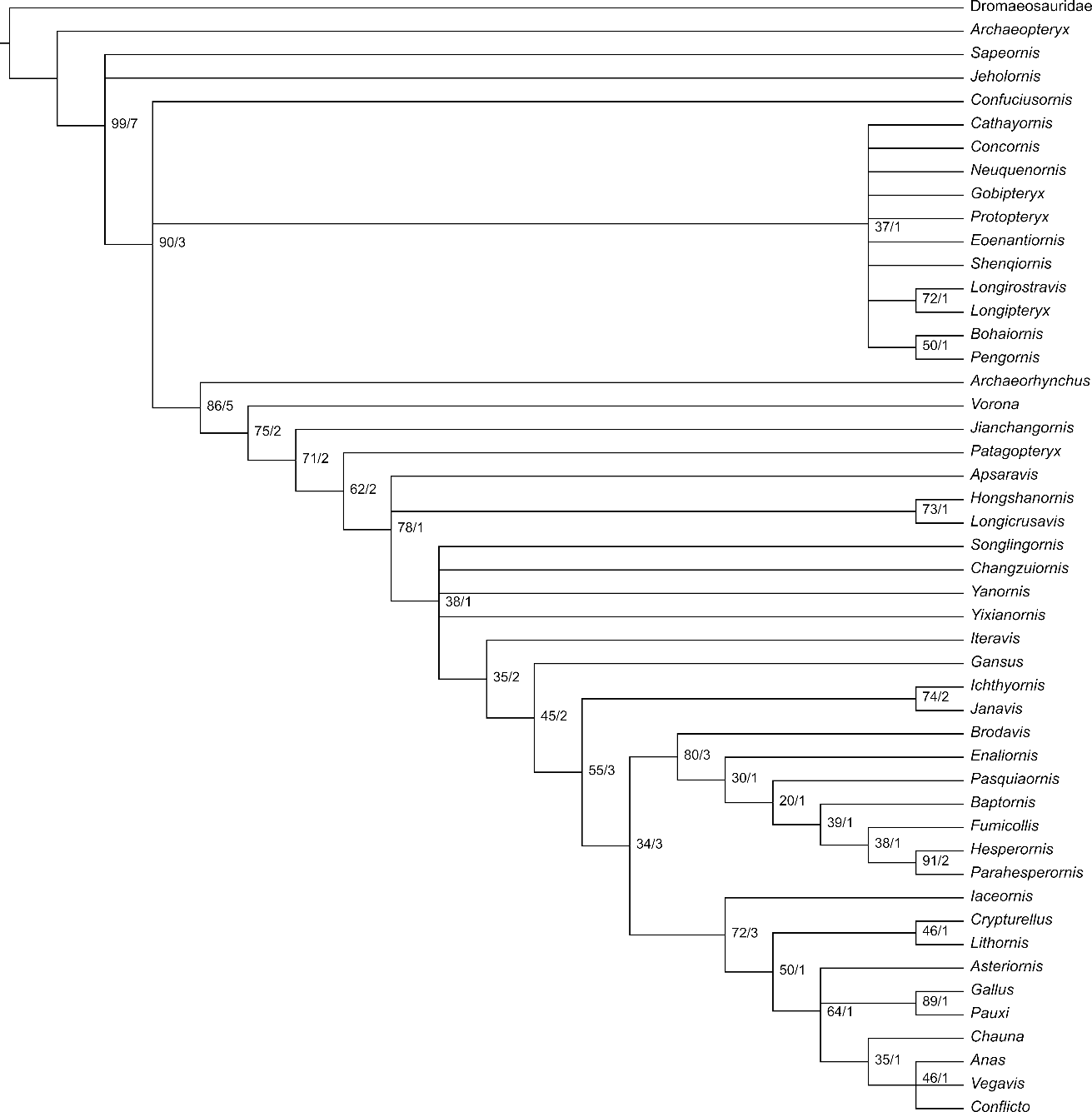


**Supplementary Figure 11.** Strict consensus tree of 11,880 most parsimonious trees recovered from the analysis of the modified dataset from Torres et al. (2021). Node values indicate bootstrap values (left) and Bremer support values (right).

**Mandibular apomorphies and character distribution**

Below is a list of the phylogenetic history and character transitions for all relevant mandibular characters across our focus clades and taxa, mapped onto the consensus tree recovered from out analyses of the modified Torres et al. (2021) dataset.

Mandibular Synapomorphies & Autapomorphies

Palaeognatahe

- **Character 42: 0 🡪 1** – Caudal end of the dentary strongly forked
- **Character 44: 0 🡪 1** – Flat to convex rostrocaudally extensive dorsal surface developed on the mandibular symphysis
- **Character 47: 1 🡪 0** – Mandibular symphyseal foramina opening on the caudal end of the symphysis

Galliformes

- **Character 47: 1 🡪 0** – Mandibular symphyseal foramina opening on the caudal end of the symphysis

*Asteriornis*

- **Character 48: 1 🡪 0** – Meckelian groove not completely covered by the splenial, deep and conspicuous medially

Mandibular character state distribution

- Character 41. Articular pneumaticity: (0) absent, (1) present.
  - Character variable across Ornithurae, articular pneumaticity present in *Ichthyornis*, absent in *Hesperornithes*.
  - Articular pneumaticity optimized as plesiomorphic for Neornithes.
  - Absence of articular pneumaticity autapomorphic for both *Gallus* and *Vegavis*.
- Character 42. Dentary strongly forked posteriorly: (0) unforked, or with a weakly developed dorsal ramus, (1) strongly forked with the dorsal and ventral rami approximately equal in posterior extent
  - Dentary exhibiting a short or no caudal fork is widespread across the tree, optimized as plesiomorphic for Ornithurae, Neornithes and Galloanseae/Neognathae.
  - The presence of a strong caudal fork is optimized as a synapomorphy of Palaeognathae
- Character 43. Splenial, anterior extent: (0) splenial stops well posterior to mandibular symphysis, (1) extending to mandibular symphysis, though non-contacting, (2) extending to proximal tip of mandible, contacting on midline.
  - A long splenial extending to the mandibular symphysis, yet not contacting, is optimized as pleiomorphic for Euornithes, Ornithurae, Neornithes and Palaeognathae
  - A short splenial stopping well caudal of the symphysis is optimized as plesiomorphic for Neognathae/Galloanserae
- Character 44. Mandibular symphysis, anteroposteriorly extensive, flat to convex, dorsal-facing surface developed: (0) absent, concave, (1) flat surface developed.
  - A concave mandibular symphysis is widespread across the tree and optimizes as plesiomorphic for Ornithurae, Neornithes and Neognatahe/Galloanserae
  - The presence of a flat dorsal symphyseal surface is optimized as a synapomorphy for Palaeognathae.
- Character 45. Mandibular symphysis, symphysial foramina: (0) absent, (1) present.
  - The presence of symphyseal foramina is optimized as plesiomorphic for Neornithes.
- Character 46. Mandibular symphysis, symphysial foramen/foramina: (0) single, (1) paired.
  - Paired symphyseal foramina are optimized as plesiomorphic for Neornithes.
  - A single symphyseal foramen is optimized as an autapomorphy of *Crypturellus.*
- Character 47. Mandibular symphysis, symphysial foramen/foramina: (0) opening on posterior edge of symphysis, (1) opening on dorsal surface of symphysis.
  - Symphyseal foramina opening on the dorsal surface of the symphysis are recovered as plesiomorphic for Neornithes and Anseriformes.
  - Symphyseal foramina opening caudally are optimized as synapomoprhic for both Palaeognathae and Galliformes.
- Character 48. Meckelian groove: (0) not completely covered by splenial, deep and conspicuous medially, (1) covered by splenial, not exposed medially.
  - A meckelian groove completely covered by the splenial is widespred across the tree and recovered as plesiomorphic for Ornithurae, Neornithes, Palaeognathae and Neognathae/Galloanserae.
  - A medially exposed, not completely covered meckelian groove is optimized as an autapomorphy of *Asteriornis*.

**Supplementary references**

Benito, J., P.-C. Kuo, K.E. Widrig, J.W.M. Jagt, and D.J. Field. 2022. Cretaceous ornithurine supports a neognathous crown bird ancestor. *Nature* 612: 100–105. doi: 10.1038/s41586-022-05445-y

Botelho, J.F., L. Ossa-Fuentes, S. Soto-Acuña, D. Smith-Paredes, D. Nuñez-León, M. Salinas-Saavedra, M. Ruiz-Flores, and A.O. Vargas. 2014. New developmental evidence clarifies the evolution of wrist bones in the dinosaur–bird transition. *PLoS Biology* 12: e1001957. doi: 10.1371/journal.pbio.1001957

Field, D.J., J. Benito, A. Chen, J.W.M. Jagt, and D.T. Ksepka. 2020. Late Cretaceous neornithine from Europe illuminates the origins of crown birds. *Nature* 579: 397–401. doi: 10.1038/s41586-020-2096-0

Hou, L., L.M. Chiappe, F. Zhang, and C.-M. Chuong. 2004. New Early Cretaceous fossil from China documents a novel trophic specialization for Mesozoic birds. *Naturwissenschaften* 91: 22–25. doi: 10.1007/s00114-003-0489-1

Hu, D., X. Xu, L. Hou, and C. Sullivan. 2011. A new enantiornithine bird from the Lower Cretaceous of Western Liaoning, China, and its implications for early avian evolution. *Journal of Vertebrate Paleontology* 31: 154–161. doi: 10.1080/02724634.2012.652321

Mayr, G. and J. Clarke. 2003. The deep divergences of neornithine birds: a phylogenetic analysis of morphological characters. *Cladistics* 19: 527–553. doi: 10.1111/j.1096-0031.2003.tb00387.x

Napoli, J.G., M. Fabbri, A.A. Ruebenstahl, J.K. O’Connor, B.-A.S. Bhullar, and M.A. Norell. 2025. Reorganization of the theropod wrist preceded the origin of avian flight. *Nature* 644: 699–705. doi: 10.1038/s41586-025-09232-3

Nesbitt, S.J. and J.A. Clarke. 2016. The anatomy and taxonomy of the exquisitely preserved Green River Formation (early Eocene) lithornithids (Aves) and the relationships of Lithornithidae. *Bulletin of the American Museum of Natural History* 406: 1–91.

O’Connor, J.K., L.M. Chiappe, C. Chuong, D.J. Bottjer, and H. You. 2012. Homology and potential cellular and molecular mechanisms for the development of unique feather morphologies in early birds. *Geosciences* 2: 157–177. doi: 10.3390/geosciences2030157

O’Connor, J., X. Wang, C. Sullivan, X. Zheng, P. Tubaro, X. Zhang, and Z. Zhou. 2013. Unique caudal plumage of *Jeholornis* and complex tail evolution in early birds. *PNAS* 110: 17404–17408. doi: 10.1073/pnas.1316979110

Tambussi, C.P., F.J. Degrange, R.S. De Mendoza, E. Sferco, and S. Santillana. 2019. A stem anseriform from the early Palaeocene of Antarctica provides new key evidence in the early evolution of waterfowl. *Zoological Journal of the Linnean Society* 186: 673–700. doi: 10.1093/zoolinnean/zly085

Torres, C.R., M.A. Norell, and J.A. Clarke. 2021. Bird neurocranial and body mass evolution across the end-Cretaceous mass extinction: the avian brain shape left other dinosaurs behind. *Science Advances* 7: eabg7099. doi: 10.1126/sciadv.abg7099

Torres, C.R., J.A. Clarke, J.R. Groenke, M.C. Lamanna, R.D.E. MacPhee, G.M. Musser, E.M. Roberts, and P.M. O’Connor. 2025. Cretaceous Antarctic bird skull elucidates early avian ecological diversity. *Nature* 638: 146–151. doi: 10.1038/s41586-024-08390-0

Wang, X., J.K. O'Connor, X. Zheng, M. Wang, H. Hu, and Z. Zhou. 2014. Insights into the evolution of rachis dominated tail feathers from a new basal enantiornithine (Aves: Ornithothoraces). *Biological Journal of the Linnean Society* 113: 805–819. doi: 10.1111/bij.12313

Wang, X., J.K. O’Connor, J.N. Maina, Y. Pan, M. Wang, Y. Wang, X. Zheng, and Z. Zhou. 2018. *Archaeorhynchus* preserving significant soft tissue including probable fossilized lungs. PNAS 115: 11555–11560. doi: 10.1073/pnas.1805803115

Zhou, S., J.K. O’Connor, and M. Wang. 2014. A new species from an ornithuromorph (Aves: Ornithothoraces) dominated locality of the Jehol Biota. *Chinese Science Bulletin* 59: 5366–5378. doi: 10.1007/s11434-014-0669-8
